# Supplementary figures and images for: Recurrent network interactions explain tectal response variability and experience-dependent behavior
Source: eLife. 2023 Mar 21;12:e78381. doi: 10.7554/eLife.78381 (PMC10030118; doi:10.7554/eLife.78381)

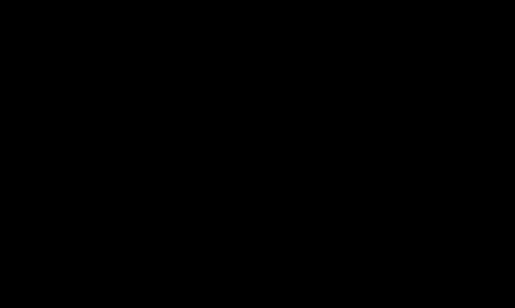

Supplement: Source data 1. — TIFF stack containing binary mask defining the tectal SPV anatomical region, in ZBB space. [file elife-78381-data1.zip › Volumetric OT SPV Maskx0.5.tif]
